# Computational derivation of a molecular framework for hair follicle biology from disease genes

**DOI:** 10.1038/s41598-017-16050-9

**Published:** 2017-11-24

**Authors:** Rachel K. Severin, Xinwei Li, Kun Qian, Andreas C. Mueller, Lynn Petukhova

**Affiliations:** 1 Department of Dermatology, College of Physicians & Surgeons, New York, NY USA; 2 Data Science Institute, Columbia University, New York, NY USA; 3 Department of Biostatistics, Mailman School of Public Health, New York, NY USA; 4 Department of Epidemiology, Mailman School of Public Health, New York, NY USA

## Abstract

Knowledge about genetic drivers of disease increases the efficiency of interpreting patient DNA sequence and helps to identify and prioritize biological points of intervention. Discoveries of genes with single mutations exerting substantial phenotypic impact reliably provide new biological insight, although such approaches tend to generate knowledge that is disjointed from the complexity of biological systems governed by elaborate networks. Here we sought to facilitate diagnostic sequencing for hair disorders and assess the underlying biology by compiling an archive of 684 genes discovered in studies of monogenic disorders and identifying molecular annotations enriched by them. To demonstrate utility for this dataset, we performed two data driven analyses. First, we extracted and analyzed data implicating enriched signaling pathways and identified previously unrecognized contributions from Hippo signaling. Second, we performed hierarchical clustering on the entire dataset to investigate the underlying causal structure of hair disorders. We identified 35 gene clusters representing genetically derived biological modules that provide a foundation for the development of a new disease taxonomy grounded in biology, rather than clinical presentations alone. This Resource will be useful for diagnostic sequencing in patients with diseases affecting the hair follicle, improved characterization of hair follicle biology, and methods development in precision medicine.

## Introduction

In an age of precision medicine, faced with interpreting DNA sequence in the genomes of patients, it becomes critical to understand both the spectrum of genes that could be contributing to a particular clinical presentation, and the pathways that are mediating genetic effects. An archive of disease genes facilitates diagnostic sequencing^1^. Rigorous analysis of the functional relationships across a set of genes linked to a particular disease state has the potential to provide robust molecular characterization of both disease pathogenesis and human physiology, and could help illuminate a causal structure that underpins health and tissue homeostasis. Such work can have a profound impact on patient care by prioritizing pathways to therapeutically target, guiding drug development, suggesting drug repurposing opportunities, and improving the efficiency of clinical trials^2^. Additionally, efforts to functionally organize disease genes would provide a foundation for the development of a new disease taxonomy that is grounded in biology, rather than clinical observations of symptoms alone. The need to develop an improved disease taxonomy by incorporating mechanistic information from molecular data has been identified as a critical challenge in the advancement of precision medicine^3^. However, such efforts have yet to be rigorously pursued in clinical areas outside of oncology.

Our knowledge of genes that influence human health and disease is largely derived from two complementary gene mapping approaches. Linkage studies and exome sequencing in families segregating rare Mendelian (i.e. monogenic) diseases have identified mutations that are rare in the population and exert strong biological effects that tend to be easy to interpret, thereby facilitating identification of a definitive causal gene and providing insight into disease mechanism. On the other hand, genome-wide association studies (GWAS) are performed in large cohorts of unrelated patients and controls and identify genetic variants with greater population frequencies. Variants identified through GWAS tend to be intergenic and have obscure biological effects, thereby hampering the definitive identification of individual causal genes. Therefore, Mendelian disease genes offer a clear advantage over GWAS loci in gaining biological insight.

Although Mendelian diseases are infrequent within the population, evidence continues to emerge from human genetic studies that etiological information derived from rare diseases caused by single mutations is sometimes generalizable to diseases that are common in the population and have a polygenic architecture^1,4–7^. Conceptually, there are a finite number of physiological processes that can drive a particular disease manifestation. If we consider a single biological pathway that contributes to homeostasis in a particular tissue (or set of tissues), there may be genes for which a single mutation exerts an extreme effect, or genes for which an accumulation of variants shifts the tissues towards a disease state. This suggests that diseases across the full spectrum of etiological heterogeneity and population prevalence could share an underlying causal structure. Examples in which gene identification in Mendelian diseases have led to new therapeutic approaches to common diseases provide the most direct evidence for a shared underlying biological architecture^1^. In further support of this theory, there is evidence that Mendelian disease genes make direct contributions to common disorders, for example when GWAS identify loci that harbor genes that cause Mendelian disorders^1,5,6^. Finally, it has been shown that deleterious variants in Mendelian loci can contribute non-additively to the risk of developing certain complex diseases affecting similar systems^5^. Therefore, we propose that constructing a molecular taxonomy from genes implicated in rare disorders could provide valuable insight into the underlying causal structure of common disorders that have clinical presentations overlapping partially with more extreme Mendelian phenotypes.

Dermatological disorders provide salient opportunities for developing methods in precision medicine. Direct visual assessment of diagnostic cues and histological findings allows for a relatively high precision in diagnoses and nuanced phenotypic subtyping. Hair disorders in particular represent a unique opportunity to develop disease taxonomies from genetic data, as gene mapping in humans and animal models has identified hundreds of genes that affect multiple aspects of hair follicle biology, including hair follicle size, density and cycling, as well as hair fiber length, shape, texture and pigmentation. Despite the tremendous amount of data generated from genetic studies of hair, and from molecular and functional studies of genes, there has yet to be a large-scale analysis to integrate all of the available information and generate new biological knowledge about genetic modulators of the hair follicle.

Here, we have curated a database of genes for which a single mutation influences hair follicle phenotypes. We identified 684 genes from publicly available resources and from literature describing single gene hair disorders in humans and mammalian models. We annotated these genes across multiple molecular and functional domains and identified 4,937 terms significantly enriched by these genes. In order to demonstrate utility for such a data set, we performed two sets of analyses. First, we extracted data pertaining to cellular signaling pathways to construct and analyze a hair follicle signaling network. Second, we performed hierarchical clustering analysis and natural language processing (NLP) to identify functional clusters of genes and describe relationships within and among these sets of genes. This work provides a valuable resource for advancing the implementation of precision medicine and may be used for diagnostic sequencing, genetic characterization of the hair follicle at an unprecedented scale, and methods development in disease taxonomy.

## Results

We identified 684 protein-coding genes that influence the integrity of the hair follicle via an inherited genetic mutation in human patients and/or mammalian models and could be mapped to unique Human Genome Organization (HUGO) gene nomenclature committee (HGNC) approved gene symbols (Supplementary Table 1). We characterized the biology implicated by these genes by performing annotation enrichment analysis, which identified 4,937 significantly enriched annotations (Supplementary Table 2), including terms descriptive of gene ontology, biological pathways, protein domains and interactions, gene expression patterns in tissues, and disease connections (Supplementary Table 3).

Several biological themes emerge from a review of the significantly enriched annotations. For example, 962 gene ontology (GO) terms are significantly enriched by 678 genes; 91 of these genes are involved in various cellular metabolic processes, including glucose and lipid metabolism; 222 influence development of organs and tissues outside of the integumentary system including heart, kidney, brain and other tissues of the nervous system, and digestive system including pancreas. Pathway analysis identified 300 pathways significantly enriched by 384 of the 684 genes, including 153 genes that enrich one or more cancer pathways, including not only melanoma and basal cell carcinoma, but also brain, pancreatic, thyroid, lung, endometrial, and colorectal cancers, among others; 134 genes enrich pathways that are annotated to be implicated in response to a viral or bacterial pathogen.

**Figure 1:**
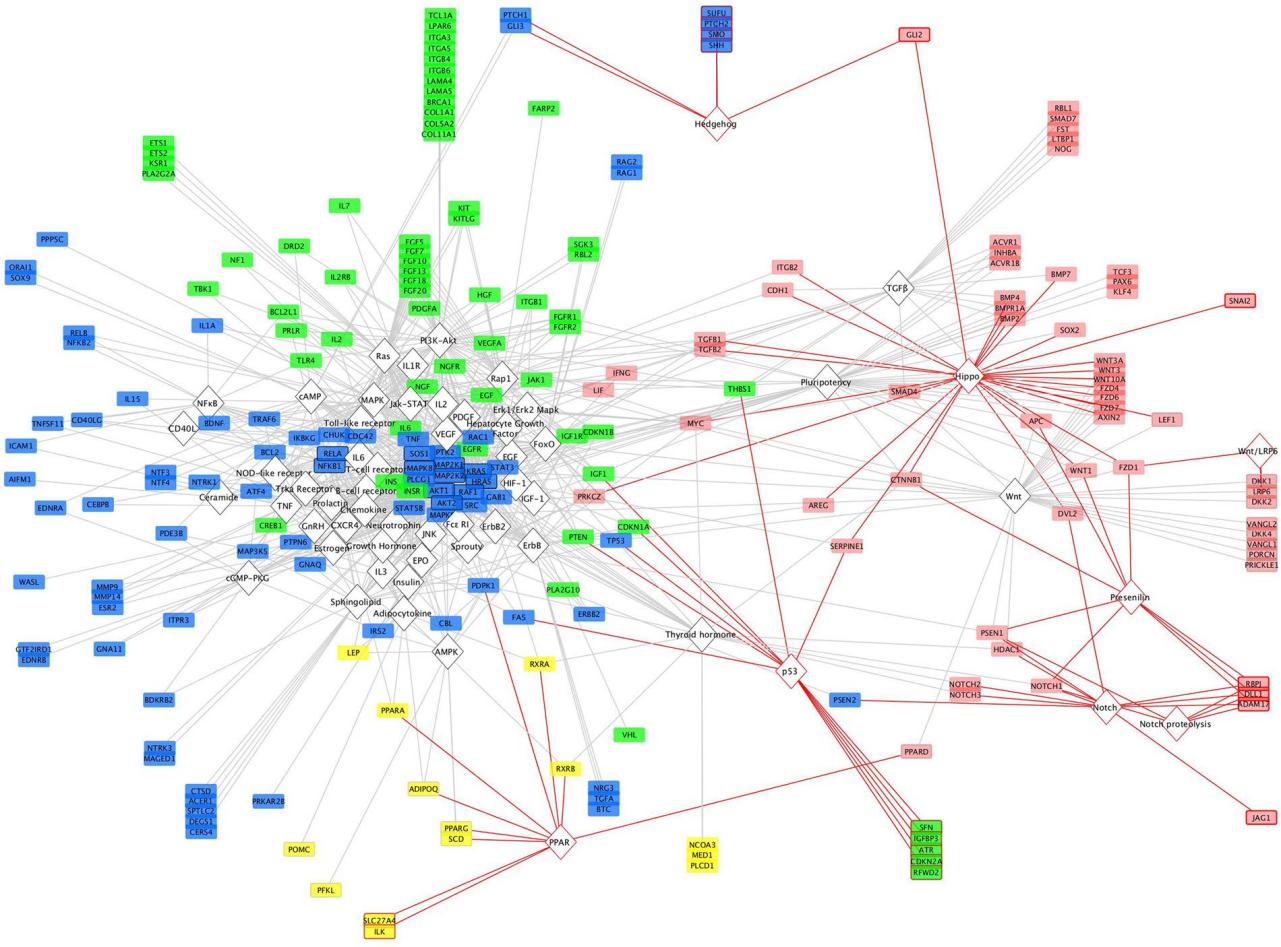
Hair follicle signaling network revealed by genes underlying monogenic disorders. Annotations significantly enriched by the 684 genes we identified include 57 cellular signaling pathways (diamond nodes) that are connected by a network of 220 genes (rectangular nodes). Edges represent gene-pathway memberships. The most highly connected genes (black outlines) connect 49 pathways (black outlines). Of the eight pathways that do not contain any of the highly connected genes (red outlines), seven are connected by a set of 59 genes (indicated by red edges). This subnetwork was also identified by the Louvain method for gene community detection (red nodes) as one of four gene communities, and includes all 29 genes of the Hippo pathway. The other three gene communities are color-coded, indicating a consistency of results across both analytic methods.

There are 57 cellular signaling pathways significantly enriched by 220 genes, including Wnt, Hippo, TGFβ, Hedgehog, Notch, PI3K-Akt, MAPK, ErbB, Ras, and JAK-STAT pathways, among others (Supplementary Table 4). An analysis of gene distributions across these signaling pathways reveals a complex network in which all pathways are linked by various subsets of genes (Fig. 1). Genes display differing levels of connectivity within this signaling network, participating in as few as one pathway (n = 70) and as many as 40 pathways (MAP2K1). The most highly connected genes in the network (n = 11), participating in 19 or more pathways each, connect 49 of the 57 pathways. Of the remaining eight pathways, which do not contain any of these highly connected genes, seven are connected by a set of 59 genes linking the Hippo pathway to Hedgehog, Wnt, Notch, and p53 signaling pathways (Fig. 1, red edges). This subnetwork is additionally identified by gene community detection (Fig. 1, red gene nodes), which identified a total of four gene groupings (modularity = 0.2346) on the basis of pathway membership (Supplementary Table 4).

**Figure 2:**
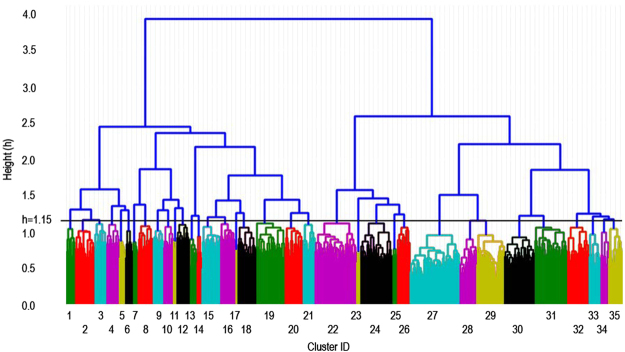
Molecular taxonomy of hair disorder genes revealed by functional hierarchical clustering analysis of 684 genes and 4,937 annotations. Unsupervised agglomerative hierarchical clustering was performed to group 684 genes based on the degree of similarity among their functional annotations. Color-coding distinguishes 35 clusters created by using an arbitrary threshold of height (h) = 1.15, indicated by a black horizontal line. Genes with similar functional annotations are grouped within the same or neighboring clusters. We propose that each cluster represents a biological module, a set of genes that converge on a shared biological feature whose diagnostic and clinical utility remain to be established.

We next performed a hierarchical clustering analysis to characterize the functional relationships among these 684 genes that are captured by significantly enriched annotations. We utilized an unsupervised agglomerative clustering algorithm to generate a dendrogram that can be used to estimate relative pairwise molecular and functional similarity between any two genes by tracing branches between them (Fig. 2; Supplementary Table 5). For example, a longer distance along branches between two genes indicates fewer similarities, and close neighbors are expected to have more annotations in common.

Finally, we sought to investigate the causal structure of hair disorders under the hypothesis that this comprehensive set of genes would converge upon a discrete number of biological processes that are critical for governing hair follicle biology. The dendrogram allows us to optimize the number of gene clusters by varying an arbitrary height threshold. Through an iterative process, we found that setting the height to h = 1.15 generated 35 semantically meaningful gene clusters. We validated this threshold with two sequential procedures. First, principal component analysis (PCA) was performed and the number of components was set to 100, which was indicated to be reasonable on the basis of variance analysis. Second, subsequent t-SNE visualization of the PCA output with 35 clusters labeled was performed, revealing adequate boundaries among clusters, reproduced at various levels of perplexity. NLP identified defining features of each cluster (Supplementary Table 6), which were used to develop semantic descriptions based on functional annotation content for each of the clusters (Table 1).

**Table 1:** Summary of natural language processing of cluster annotations.

Cluster	Gene Count	Mapped Genes	Term Extraction
1	11	11	choline metabolism in cancer, binding site:atp, kinase, hsa04722:neurotrophin signaling pathway, hsa04071:sphingolipid signaling pathway, hsa04910:insulin signaling pathway
2	24	23	pi3k-akt signaling pathway, hsa04014:ras signaling pathway, kinase
3	15	15	hsa05100:bacterial invasion of epithelial cells, hsa04520:adherens junction, hsa04510:focal adhesion
4	15	15	hsa04110:cell cycle, 7157:tp53tumor protein p53, heat shock protein, nucleolin
5	8	8	obesity, dna-binding region:nuclear receptor, steroid hormone receptor
6	8	8	NKκB signaling pathway
7	7	7	autoimmune disease, infection, graft-versus-host disease
8	19	18	cardiovascular diseases, autoimmune disease, atherosclerosis, obesity, metabolic syndrome, type 2 diabetes
9	13	13	T-cell factor dependent signaling, hormone
10	12	12	lysosome, lysosomal lumen, glycosaminoglycan degradation
11	4	4	synaptic vesicle transport, melanosome organization, lysosomal organelles biogenesis
12	15	14	keratinocyte differentiation, foreskin
13	10	10	keratin, intermediate filament, ipr003054:type ii keratin
14	6	6	keratin, intermediate filament, ipr002957:keratin type i
15	23	21	magnesium, protein heterooligomerization
16	18	17	cell differentiation, fatty acid biosynthesis, iron, go:0030148 sphingolipid biosynthetic process
17	3	3	ribosomal protein
18	22	22	cell-cell adherens junction, methylation, gaba type a receptor associated protein like
19	36	36	go:0045892 negative regulation of transcription dna-te, 3065:hdac1histone deacetylase 1, domain:leucine-zipper, ipr011598:myc-type basic helix-loop-helix (bhlh) domain
20	22	21	chromatin regulator, 3066:hdac2histone deacetylase 2, go:0006310 dna recombination
21	15	15	go:0007568 aging, hsa04913:ovarian steroidogenesis, iron
22	51	41	cytoplasmic vesicle, endosome, go:0000139 golgi membrane
23	5	5	go:0004713 protein tyrosine kinase activity, go:0008543 fibroblast growth factor receptor signaling, go:0036092 phosphatidylinositol-3-phosphate biosynthesic process
24	39	39	go:0042438 melanin biosynthetic process, go:0033162 melanosome membrane, go:0043066 negative regulation of apoptotic process
25	6	6	go:0030057 desmosome, ipr014868:cadherin prodomain, ipr027397:catenin binding domain
26	16	16	go:0032496 response to lipopolysaccharide, myocardial infarction, go:0006954 inflammatory response
27	61	53	go:0007399 nervous system development, lipoprotein, cell projection
28	21	18	homeobox, go:0001942 hair follicle development
29	34	32	5914:retinoic acid receptor alpha(rara), cross-link:Glycyl lysine isopeptide (Lys-Gly) (interchain with G-Cter in SUMO), dna-binding, transcription regulation
30	37	35	go:0005887 integral component of plasma membrane, calcium transport, go:0043588 skin development
31	40	31	go:0043473 pigmentation
32	27	27	go:0007155 cell adhesion, go:0030198 extracellular matrix organization, go:0005788 endoplasmic reticulum lumen
33	14	14	ipr001881:egf-like calcium-binding, ipr009030:insulin-like growth factor binding protein, n terminal
34	10	10	hsa04550:signaling pathways regulating pluripotency of stem cells, hsa05205:proteoglycans in cancer, hsa04390:hippo signaling pathway, hsa04916:melanogenesis, wnt signaling pathway
35	17	16	go:0005125 cytokine activity, sm00204:tgfb, growth factor, go:0008285 negative regulation of cell proliferation

NLP identified the most frequent significantly enriched annotations specific to each of the 35 clusters, allowing for semantic interpretation of the hierarchical clustering analysis. Mapped genes indicate the number of genes annotated by at least one NLP feature. Dominant features of clusters suggest the functional significance of modules revealed by our analytic approach. In order to increase specificity of terms, annotations that appeared in more than 21 clusters (60%) were excluded from NLP. A list of the 20 most enriched annotations for each cluster may be found in Supplementary Table 6.

## Discussion

Perturbations in hair follicle biology are manifested in multiple ways, for example interrupting a developmental process leading to hair loss, or affecting the integrity of the hair fiber leading to a change in length, texture or pigmentation. Since one of our goals was to construct a resource that could be used to provide a comprehensive assessment of the biology of this organ, we set out to identify monogenic hair genes regardless of their specific phenotypic consequences. We identified 684 protein-coding genes that alter the hair follicle in human patients and/or mammalian models, representing the most comprehensive archive of hair follicle genes identified in genetic mapping experiments to date (Supplementary Table 1). This resource will be useful for constructing filtering algorithms for human genome sequence data generated for diagnostic or investigative purposes. While this set of genes is larger than has previously been reported in reviews of hair follicle genetics in humans or mouse models (e.g.^8,9^), as a reference point, gene expression experiments in human and murine models report differential expression of thousands of genes in the hair follicle (e.g.^10,11^). In order to be comprehensive, we included genes identified in either human or animal studies. We included genes that have only been characterized in animal models because of the possibility that they do contribute to human traits, but have not yet been identified in patients. For example, animal studies had identified fibroblast growth factor 5 (FGF5) as a crucial regulator of hair growth two decades before it was found to underlie a human condition^12,13^. If this gene list is incorporated into algorithms designed for filtering human DNA sequence data, we recommend including animal model genes and down-weighting evidence scores.

The genes we identified significantly enrich a set of 4,937 annotations, which provide insight into genetic regulators of hair follicle biology and relationships among disease genes (Supplementary Table 2). For example, the metabolic demands of the hair follicle dramatically increase during the growth stage of the hair cycle (anagen) to support the extensive cell proliferation and differentiation that occurs as the organ regenerates, transitioning from a quiescent state. It has been shown previously that glucose is a substantial source of energy in the growing hair follicle, which utilizes aerobic glycolysis^14,15^. We identified 40 genes annotated by glucose regulation. Likewise, lipid homeostasis is known to be important for maintaining a healthy hair follicle through the identification of several genes in various hair loss disorders^16^. Our analysis identified a total of 36 genes annotated to be involved with lipid metabolism. Other metabolic pathways implicated in hair follicle biology by our analysis include proteoglycan metabolism (four genes), cellular amino acid metabolism (six genes), and vitamin metabolism (three genes).

Our analysis identified multiple significantly enriched cancer pathways. Limitations of our analytic approach prevent us from inferring a relationship between hair disorders and cancers. Rather, the presence of cancer annotations could simply reflect the critical role that regulation of cell proliferation plays in hair follicle biology, given that this organ undergoes cycling between regeneration and regression throughout the lifespan. Likewise, annotations that implicate tissues outside of the integumentary system may be capturing shared biology or developmental lineage, and/or could be indicative of multisystem disease, but further studies are needed to understand these relationships as well.

While roles for several cellular signaling pathways in hair biology have been previously established, our work provides a comprehensive overview of how these pathways are genetically linked in the hair follicle, revealing important inflection points that await further investigation, including the identification of a new pathway for prioritization in studies focused on modulating hair growth. Previously, the WNT pathway has been shown to be a critical regulator of hair follicle development and cycling, and roles for TGFβ, Notch, Hedgehog and JAK-STAT signaling have also been established^17,18^. Our network analysis not only demonstrates contributions from these pathways in causing genetic perturbations in the hair follicle, but also implicates Hippo signaling through the identification of 29 genes annotated to participate in this pathway (Fig. 1; Supplementary Table 4). While Hippo signaling has been previously studied in the skin, specifically in mouse epidermal development and human cutaneous squamous cell carcinoma^19,20^, the pathway has yet to be investigated as a potential modulator of the hair follicle.

Hippo signaling has been extensively studied in the contexts of cancer and development, and has been shown to influence tumor or organ size through the regulation of cell proliferation and apoptosis^21,22^. In fact, the pathway was originally given its name because genetic perturbations thereof generated “hippopotamus-sized” organs^22^. Interestingly, the most common form of hair loss, androgenetic alopecia (i.e. male pattern baldness; MPB), has long been characterized as a process of organ miniaturization, whereby hair follicles continue to cycle but undergo a reduction in size, resulting in a transition from thick terminal hair to fine vellus hair^23^. While Hippo signaling has yet to be specifically implicated in MPB, there is preliminary genetic evidence that is consistent with such a hypothesis. The largest MPB GWAS performed to date included a gene-based analysis that identified 112 autosomal genes with genome-wide significant association (Bonferroni correction of α < 2.769e-06)^24^, four of which are annotated to participate in Hippo signaling within the Kyoto Encyclopedia of Genes and Genomes (KEGG; pathway hsa04390), including WNT6, WNT10A, WNT3, and CTNNB1. We performed pathway enrichment analysis of these 112 genes and identified hsa04390:Hippo signaling pathway at a significance level of p = 0.049. Three of these genes reside at loci that were also associated with MPB in an independent GWAS (WNT6, WNT10A, WNT3)^25^. Our analysis of genes that establish a hair follicle cellular signaling network identified the Hippo pathway and a set of 59 genes that link this pathway to Wnt, Notch, Hedgehog and p53 signaling pathways (Fig. 1). A definitive role for Hippo signaling in the pathogenesis of MPB awaits further investigation.

In order to characterize relationships among the 684 genes that influence hair follicle biology through single mutations, we used the set of 4,937 significantly enriched annotations to perform hierarchical clustering. We identified an organizational scheme derived from functional and molecular data, and thus rooted in biology (Fig. 2; Supplementary Table 5). As a preliminary strategy to understand the biological structure suggested by this clustering, we defined 35 gene clusters by optimizing a height threshold (h = 1.15) and using NLP to identify biological themes within clusters (Table 1). We propose that each cluster represents a biological module, a set of genes that converge on a shared biological feature whose diagnostic and clinical utility remain to be established. We found, for example, that Cluster 7 represents a set of genes annotated by terms related to autoimmune disease and pathogen response, and contains a number of genes that mediate tissue interactions with the immune system, including INFG, IL2, IL2RB, and FAS. This supports recent work that has implicated the immune system in hair follicle development^26^, and suggests further investigation into roles that the immune system may play in hair follicle cycling and homeostasis is warranted.

While our work provides a framework for understanding the biology that influences hair follicle disease, future work linking the biological modules that we identified to disease phenotypes will help to better understand the complex relationship between molecular functions of genes and the disease that arise from mutations in them. There is preliminary evidence that our gene clustering may have diagnostic relevance. For example, cluster 18 is enriched with annotations such as “cell-cell adherens junction” and contains genes that code for components of cellular anchoring junctions. Disruptions in these proteins produce multi-system clinical manifestations that include hypotrichosis and/or woolly hair^27^. Mutations in Plakophilin 1 (*PKP1*) cause an inherited disease impacting ectodermal structures, and patients display hypotrichosis, nail dystrophy, and skin fragility^9^. Mutations in junctional plakoglobin (*JUP*) and desmoplakin (*DSP*) cause Naxos disease and Carvajal syndrome respectively, two cardiocutaneous syndromes that include symptoms of woolly hair, cardiomyopathy, and palmoplantar keratoderma^9^. Our analysis places these three genes adjacent to each other in cluster 18, seemingly capturing biological similarities among disease entities with partially overlapping phenotypes.

Alternatively, some clustering results suggest that there may be degenerate mapping between clinical symptoms and molecular or functional characterization of disease genes. For example, uncombable hair syndrome is a nonsyndromic hair disorder with three recently identified causative genes: tricohyalin (*TCHH*), transglutaminase 3 (*TGM3*), and peptidylarginine deiminase 3 (*PADI3*)^28^. Our hierarchical clustering analysis placed *TGM3* and *TCHH* in cluster 22, whereas PADI3 is in cluster 31. An analysis of annotations that are significantly enriched by these three genes suggests that it is the distribution of transcription factor binding sites that is driving this distinction (Supplementary Table 7). Interestingly, these genes show different patterns of gene expression in The Genotype-Tissue Expression (GTEx) database^29^, which suggests differences in regulatory elements. While further investigation is required to determine if these results have clinical relevance, this example does suggest possible biological distinctions between diseases that are traditionally grouped together as a single entity on the basis of symptoms, providing motivation for further development of a disease taxonomy that incorporates data from molecular biology experiments. Future work should focus on integrating and evaluating clinical manifestations with molecular annotations.

The goal of clustering methods is to find structure within data, grouping similar elements within the same cluster and dissimilar elements in different clusters. For example, our clustering model separates genes encoding type II (basic) keratins (cluster 13) and type I (acidic) keratins (cluster 14) into adjacent clusters, capturing both their similarities and differences by their relative dendrogram positions. However, as with any unsupervised machine learning method, analytic outcomes may be influenced by the data available for input and the choice of algorithms used to determine similarities among elements (in this case, genes). For example, we annotated genes with functional and molecular data that is currently available in the public domain and integrated into pathway analysis software^30^. Experimental data that continues to accumulate over time could influence clustering results. Furthermore, there are a number of algorithms available to uncover structure in data. We applied unsupervised agglomerative hierarchical clustering, which required us to empirically determine an optimal number of clusters within the data set. We used an iterative process evaluating and integrating NLP results to partition our dendrogram into 35 clusters, obtained by setting a height threshold of h = 1.15. We believe this to be an optimal operationalization of causal structure because it generated semantically meaningful clusters with NLP. Additionally, while the dimensionality reduction method that we employ has been widely adopted for deriving meaning from high-dimensional data^31^, interpretation of results has some inherent challenges. For example, the algorithm adapts to the underlying data, performing different translations on different regions of data, which may present a source of confusion in visual interpretation^32^. The analysis that we report here is presented as an example of the diverse analytic approaches that could be applied to this Resource in future investigations.

Our work in identifying and functionally annotating a comprehensive set of genes that underlie hair disorders provides a valuable resource for both research and clinical communities embarking on precision medicine initiatives for skin and hair disorders, and could be useful for methods development more broadly relevant to the implementation of precision medicine across other clinical areas. Understanding disease causation in patients and devising efficient therapeutic strategies requires knowledge not only of the genes implicated in disease, but also of their interactions through biological pathways, which may reveal a higher order causal structure of disease^4^. This archive provides a tool for pinpointing loci harboring critical mutations that underlie diseases with clinical manifestations in the hair follicle, and for surveying pathways and biological processes that modulate the hair follicle. We have utilized analytic approaches drawn from the field of machine learning in an initial attempt to functionally link genes based on biological knowledge that is currently available in the public domain, uncovering insight into physiology that is critical to hair biology and disease. Our work invites prioritization of the Hippo signaling pathway in future studies of molecular modulation of hair growth and has identified higher order biological structure among these 684 genes. This work creates an opportunity for future methods development in precision medicine.

## Methods

### Identification of genes

We compiled a genotype-phenotype database incorporating genes from two publicly available data sources, Online Mendelian Inheritance in Man (OMIM) catalog and Jackson Laboratories Mouse Genome Informatics (MGI) database, as well as human and mammalian model studies from the literature (Supplementary Table 1).

A preliminary list of human genes influencing hair phenotype was created using a series of phenotype searches within OMIM. We defined the following 7 categories to characterize hair phenotype: alopecia, hair cycling, hypertrichosis, hair morphogenesis, hair pigmentation, hair structure, and secondary effects on hair. “Secondary effects” refers to alterations in hair phenotype secondary to a primary alteration in metabolic phenotype. We reduced the risk of false-negative search results by using multiple synonymous descriptors as search terms in OMIM (Supplementary Table 8). We mitigated the risk of false-positive search results by excluding genes that were annotated in OMIM to be without a known gene sequence, and/or with a provisional relationship with the disease, and/or without a gene map locus. Corresponding search terms were used to identify mouse genes with human orthologs linked to hair phenotypes within the MGI database. A list of additional genes known to influence hair phenotype in humans and other mammalian models, including mouse, rat, dog, and horse, was compiled from reports in the literature^28,33–39^. We next excluded genes that are not protein-coding and/or do not have human orthologs, removing pseudogenes, heritable phenotypic markers, quantitative trait loci, chromosomal inversions, transgenic mutations that implicated multiple genes, and polygenic mutations. Gene symbols were standardized to HGNC-approved gene symbols for subsequent annotation and analysis.

### Gene annotation

The list of official gene symbols was uploaded to the functional annotation tool on the Database for Annotation, Visualization and Integrated Discovery (DAVID) v.6.8. Species and background were set to “Homo sapiens.” Queried categories of annotations are listed in Supplementary Table 3. Functional annotations for which p < 0.05 were downloaded to an Excel database (Excel 2016, Microsoft Corp, Seattle, WA). Significantly enriched annotations that were molecularly uninformative were removed (“disease mutation”, “polymorphism”, and “sequence variant”). To prepare the data for functional hierarchical clustering, a binary matrix of annotations for the set of genes was created in Excel.

### Signaling network construction

Significantly enriched pathways that contain the term “signaling” were extracted from the gene annotation database (Supplementary Table 2). Pathway names and gene names were imported to Cytoscape v.3.4.0 (Supplementary Table 4) to construct a network with the edge-weighted spring embedded layout^40^. Highly connected genes are defined as being within the 95^th^ percentile of pathway connections, which was empirically determined to be participating in more than 16 pathways (Supplementary Figure 1).

### Gene community detection

To identify communities of genes with similar pathway membership we used the Louvain method as implemented in R 3.3.3 using the igraph package, first constructing an adjacency matrix from Supplementary Table 4 in R^41^.

### Hierarchical clustering

Subsequent analyses were performed using Python in Jupyter Notebook, using the numpy, sklearn, pandas, and SciPy packages, as well as matplotlib and Seaborn visualization libraries. Dimensionality reduction was performed using the principal component analysis function from sklearn with the number of components set to 100, followed by visualization using t-distributed stochastic neighbor embedding (t-SNE) via the TSNE function from sklearn, with perplexity values in the range (5–250). A pairwise distance matrix was created using the pdist function in the scipy.spatial.distance module in Python, applying the Jaccard metric. Unsupervised agglomerative hierarchical clustering was performed with the linkage function in the scipy.cluster.hierarchy module, with method set to Ward (i.e. Ward variance minimization). The output was plotted with the Seaborn visualization library in Python. Several iterations of NLP (see below) were performed at various height thresholds partitioning the dendrogram into different numbers of clusters. An arbitrary height threshold of 1.15 was set to partition the dendrogram into a set of 35 clusters, which was found to yield semantically meaningful results from NLP.

### Natural language processing

NLP using pandas and numpy packages and matplotlib plotting library in Python identified the most frequent annotations associated with each cluster. All significantly enriched annotations that appeared in fewer than 60% of the clusters (n ≤ 20) were used for the analysis. Weights were assigned based on the relative frequency of a given annotation across clusters, to preferentially down-weight common annotations (Supplementary Table 5). The relative weights were defined as the count of a given annotation in a given cluster, multiplied by the natural log of the inverse quotient of the count of that annotation across all clusters divided by the total count of all annotations across all clusters. This allowed for the rational development of semantic descriptions of clusters, derived from frequent annotations associated with a given cluster (Table 1).

## Electronic supplementary material

Supplementary Information

Dataset 1

Dataset 2

Dataset 3

Dataset 4

Dataset 5

Dataset 6

Dataset 7

Dataset 8
