# Supplementary material for: Computational derivation of a molecular framework for hair follicle biology from disease genes
Source: Sci Rep. 2017 Nov 24;7:16303. doi: 10.1038/s41598-017-16050-9 (PMC5701154; doi:10.1038/s41598-017-16050-9)
Supplement: Supplementary file 1 — Supplementary Information [file 41598_2017_16050_MOESM1_ESM.pdf]

Computational derivation of a molecular framework for hair follicle biology from disease genes

Rachel K. Severin[1], Xinwei Li[2], Kun Qian[3], Andreas C. Mueller[2], and Lynn Petukhova[1,4]

1. Department of Dermatology, College of Physicians & Surgeons, New York, NY, USA
2. Data Science Institute, Columbia University, New York, NY, USA
3. Department of Biostatistics, Mailman School of Public Health, New York, NY, USA
4. Department of Epidemiology, Mailman School of Public Health, New York, NY, USA

Address for correspondence
Lynn Petukhova, PhD
Columbia University
Department of Dermatology
College of Physicians & Surgeons
Department of Epidemiology
Mailman School of Public Health
Russ Berrie Medical Science Pavilion
1150 St. Nicholas Ave, Room 303
New York, NY 10032
Phone: 212-851-4839
Fax: 212-851-1032
Email: lynn.petukhova@columbia.edu

Supplementary Figures

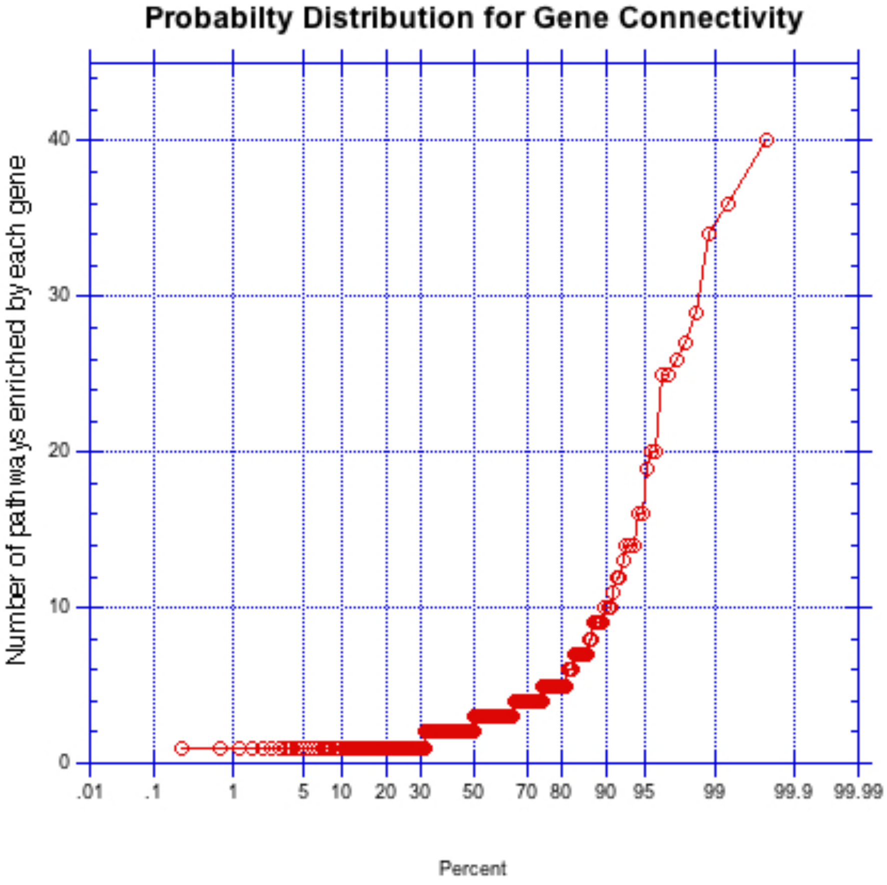

Supplementary Figure 1. Probability distribution of gene connectivity in the hair follicle signaling network. For each gene, the number of network pathways to which it belongs was summed and ranges from 1 to 40. The probability distribution indicates that the 95% percentile includes genes that belong to more than 16 pathways, defining a set of 11 genes that are highly connected within the network. Each dot represents one of 220 genes that comprise the network.

Supplementary Table Legends

Supplementary Table 1. Genes for which single mutations in humans or mammalian models influence the hair follicle. A list of genes influencing hair phenotype in humans and mice was created using a series of phenotype searches within OMIM and MGI databases, respectively. Additional genes known to influence hair phenotype in humans and other mammalian models was compiled from reports in the literature. Gene symbols were standardized to HGNC approved gene symbols. Columns contain HGNC gene symbol, gene name, chromosome number, start and stop position

(Hg38 reference genome), source (either database or PubMed ID for article), and search term(s) used to retrieve genes. Additional information about search terms may be found in Supplementary Table 8.

Supplementary Table 2. Annotations that are significantly enriched by Mendelian genes that influence the hair follicle. A list of 684 genes was imported into the functional annotation tool on the Database for Annotation, Visualization and Integrated Discovery (DAVID) v. 6.8., and a list of functional annotations significantly enriched by those genes was generated. The output from DAVID is listed here. Columns contain DAVID annotation category, term (annotation), count of genes enriching that annotation which are in the list of 684 genes, list total, percentage, P value, and HGNC gene symbol.

Supplementary Table 3. Annotation types queried in DAVID. The list of official gene symbols was uploaded to the functional annotation tool on the Database for Annotation, Visualization and Integrated Discovery (DAVID) v. 6.8. Species and background were set to "Homo sapiens." This is a list of annotation types and categories queried, along with counts of terms within those categories and the number of genes enriching those terms. Columns contain annotation type, categories, and terms.

Supplementary Table 4. Significantly enriched signaling pathways and Mendelian genes that contribute to enrichment. Data was imported into Cytoscape to generate Figure 1. Columns contain enriched term, corresponding pathway identity, HGNC gene symbol, and the results of gene community detection performed with the Louvain method.

Supplementary Table 5. Results of Hierarchical Clustering. Hierarchical clustering results with threshold of h=1.15, generating 35 clusters, are listed. Columns contain HGNC gene symbol, official gene name, dendrogram position, and cluster ID.

Supplementary Table 6. Results of natural language processing to assign annotation themes to clusters. Natural language processing (NLP) using pandas and numpy packages and matplotlib plotting library in Python identified the most frequent annotations associated with each cluster. All significantly enriched annotations that appeared in twenty or fewer clusters (60% of clusters) were used for the analysis. Among the remaining annotations, those frequent across all clusters were down-weighted. Within-cluster weights are defined as the count of an annotation withinin a given cluster, multiplied by the natural log of the inverse of the quotient of the count of that annotation across all clusters, divided by the total count of all annotations across all clusters. Columns contain cluster ID, annotation, and weighted within-cluster count.

Supplementary Table 7. An analysis of annotations enriched by TCHH, TGM3, and PADI3, causative genes associated with uncombable hair syndrome. Uncombable hair syndrome is a non-syndromic hair disorder with three recently identified causative genes: tricohyalin (*TCHH*), transglutaminase 3 (*TGM3*), and peptidylarginine deiminase 3 (*PADI3*) Our hierarchical clustering analysis placed *TGM3* and *TCHH* in cluster 22, whereas PADI3 is in cluster 31. An analysis of annotations that are significantly enriched by these three genes suggests that it is the distribution of transcription factor binding sites that is driving this distinction. Columns contain category, type, enrichment of PADI3 (cluster 22), TGM3 (cluster 22), and TCHH (cluster 31), and total counts.

Supplementary Table 8. OMIM search terms. 7 primary categories were defined to characterize hair phenotype: alopecia, cycling, hypertrichosis, morphogenesis, pigmentation, structure, and secondary effects. We reduced the risk of false-negative search results by using additional related search terms in OMIM. Columns contain primary category and additional OMIM search terms.
